# Lifestyle and behavioral factors and mitochondrial DNA copy number in a diverse cohort of mid-life and older adults

**DOI:** 10.1371/journal.pone.0237235

**Published:** 2020-08-12

**Authors:** Chirag M. Vyas, Soshiro Ogata, Charles F. Reynolds, David Mischoulon, Grace Chang, Nancy R. Cook, JoAnn E. Manson, Marta Crous-Bou, Immaculata De Vivo, Olivia I. Okereke

**Affiliations:** 1 Department of Psychiatry, Massachusetts General Hospital and Harvard Medical School, Boston, Massachusetts, United States of America; 2 Channing Division of Network Medicine, Department of Medicine, Brigham and Women’s Hospital and Harvard Medical School, Boston, Massachusetts, United States of America; 3 Department of Psychiatry, UPMC and University of Pittsburgh School of Medicine, Pittsburgh, Pennsylvania, United States of America; 4 Department of Psychiatry, VA Boston Healthcare System, Brockton, Massachusetts, United States of America; 5 Division of Preventive Medicine, Department of Medicine, Brigham and Women's Hospital and Harvard Medical School, Boston, Massachusetts, United States of America; 6 Department of Epidemiology, Harvard T. H. Chan School of Public Health, Boston, Massachusetts, United States of America; 7 IMIM (Hospital del Mar Medical Research Institute), Barcelona, Spain; 8 Centro de Investigación Biomédica en Red de Fragilidad y Envejecimiento Saludable (CIBERFES), Madrid, Spain; University of Glasgow, UNITED KINGDOM

## Abstract

Mitochondrial DNA copy number (mtDNAcn) is a putative biomarker of oxidative stress and biological aging. Modifiable factors, including physical activity (PA), avoidance of heavy alcohol use and smoking, and maintaining good mental health, may reduce oxidative stress and promote healthy aging. Yet, limited data exist regarding how these factors are associated with mtDNAcn or whether age, sex or race/ethnicity moderate associations. In this cross-sectional study, we selected 391 adults (183 non-Hispanic White, 110 Black and 98 Hispanic; mean = 67 years) from the VITAL-DEP (VITamin D and OmegA-3 TriaL-Depression Endpoint Prevention) ancillary to the VITAL trial. We estimated associations between lifestyle and behavioral factors (PA, alcohol consumption, cigarette smoking and depression) and log-transformed mtDNAcn using multivariable linear regression models. MtDNAcn was not correlated with chronological age; women had ~17% higher mtDNAcn compared to men. There were no significant associations between PA measures (frequency, amount or intensity) or alcohol consumption with mtDNAcn. Cigarette smoking (per 5 pack-years) was significantly associated with mtDNAcn (percent difference = -2.9% (95% confidence interval (CI) = -5.4%, -0.4%)); a large contrast was observed among heavy vs. non-smokers (≥30 vs. 0 pack-years): percent difference = -28.5% (95% CI = -44.2%, -8.3%). The estimate of mtDNAcn was suggestively different for past vs. no depression history (percent difference = -15.1% 95% CI = -30.8%, 4.1%), but this difference was not statistically significant. The association between smoking and log-mtDNAcn varied by sex and race/ethnicity; it was stronger in men and Black participants. While chance findings cannot be excluded, results from this study support associations of smoking, but not chronological age, with mtDNAcn and suggest nuanced considerations of mtDNAcn as indicative of varying oxidative stress states vs. biological aging itself.

## Introduction

Lifestyle and behavioral factors have been related to overall health in aging as well as to indicators of biological aging [1, 2]. Emerging evidence has linked mitochondrial dysfunction to a range of age-related diseases [3]. Lower mtDNA copy number (mtDNAcn) measured in peripheral blood cells has been associated with various adverse health outcomes in aging, including cardiovascular disease (CVD) [4], neurocognitive disorders [5] and all-cause mortality [6]. Further, oxidative stress and damage appear to precipitate changes in mitochondrial DNA (mtDNA) that are associated with mitochondrial dysfunction [7]. Thus, mtDNAcn may serve as a marker of oxidative stress as well as biological aging.

Associations between lifestyle/behavioral factors and biomarkers of molecular stress and biological aging, such as DNA methylation age and telomere length, have been reported in prior work involving large cohorts of hundreds or thousands of participants [2, 8, 9]. However, data are sparse regarding associations of modifiable factors with mtDNAcn. For example, physical activity (PA) is thought to mitigate accelerated biological aging, in part by reducing oxidative stress and inflammation. Yet, little is known about how the total amount or frequency of PA relates to mtDNAcn, or whether the type of PA (e.g., low vs. vigorous intensity activities) influences mtDNAcn. Heavy alcohol consumption and cigarette smoking appear to be instigators of oxidative stress; one mechanism may involve reduction of peroxisome proliferator-activated receptor gamma coactivator 1-alpha (PGC-1α), a translational coactivator involved in regulation of energy metabolism genes and mitochondrial biogenesis [10, 11]. In one study among a large sample of community-dwelling mid-life and older women, higher levels and duration of smoking were inversely associated with mtDNAcn [12]. However, associations of alcohol and smoking with mtDNAcn have been infrequently investigated to date. Recent reports from smaller samples (n~100) suggest associations of depression and psychosocial stress with mitochondrial changes [13, 14]. However, few studies [15, 16] have addressed associations of mood or depression with mtDNAcn in larger, community-based samples of mid-life and older men and women, and findings regarding the association are inconclusive.

Moreover, there are limited data regarding variations by race/ethnicity in the associations of lifestyle and behavioral factors with mtDNAcn or other molecular markers. Yet, the persistence of racial/ethnic disparities in healthy aging [17] and well-being highlights the importance of addressing whether such variations may exist. Additionally, prior reports [18, 19] suggest that changes in both mtDNA quantity (i.e., copy number) and quality (heteroplasmy) are independently associated with aging and disease burden. However, given the complexity of the dynamics that regulate mtDNA, the relationship between mtDNAcn and biological aging itself remains unclear. Other biomarkers of molecular stress and biological aging, such as DNA methylation age and telomere length shortening, are strongly associated with chronological age; however, correlations between mtDNAcn and chronological age have been less consistent [20–23].

The primary objective of this study was to determine the associations of key modifiable lifestyle and behavioral factors–i.e., physical activity, habitual alcohol use, cigarette smoking and mood symptoms and depression history–with mitochondrial DNA copy number in a diverse sample of nearly 400 community-dwelling mid-life and older adults. Further, we addressed whether any observed associations varied by age, sex or race/ethnicity.

## Materials and methods

### Source of participants

We selected participants from VITAL-DEP (VITamin D and OmegA-3 TriaL-Depression Endpoint Prevention, NCT01696435) [24], a late-life depression prevention ancillary study to the VITAL (NCT01169259) trial [25, 26]. VITAL consists of 25,871 men and women, aged 50+ and 55+ years (mean = 67 years), respectively, in a 2x2 factorial randomized trial of cancer and CVD prevention testing vitamin D and/or marine omega-3 (fish oil) supplementation; cohort details are provided elsewhere [25, 26].

### Sample selection

Initial selection included 300 participants in VITAL-DEP (100 non-Hispanic White, 100 Black and 100 Hispanic). Participants were balanced by age (10-year groups above age 50, where age categories followed the overall age distribution) and sex; race/ethnicity was self-reported on VITAL questionnaires. Later, a separate sample of 150 participants (50 with no prior psychiatric history, 50 with past depression and 50 with current depression/psychiatric disorder) were again balanced by age and sex tiers but not selected on race/ethnicity; this latter group of 150 participants are members of the VITAL Clinical Translational Science Center (CTSC) sub-cohort (n = 1,054), who presented for in-person baseline and follow-up visits, and also completed the VITAL-DEP CTSC protocol that featured psychiatric diagnostic interviews, as detailed elsewhere [24]. We focused on the three abovementioned racial/ethnic groups, as our study was conducted in the context of addressing potential health disparities affecting Black and Hispanic, compared to non-Hispanic White, adults. Samples from all 450 participants were sent for mtDNAcn assay and measured in a single batch. There were 445 participants who had non-missing mtDNAcn data (n = 5 lacked adequate genomic DNA available for PCR assay or had mtDNAcn below the level of detection). Participants with extreme/outlying values of mtDNAcn (n = 6), other or unknown race/ethnicity (n = 16), or missing self-reported information on physical activity, alcohol, smoking, or depression measures (n = 32) were excluded (S1 Fig). For depression measures, we excluded participants who had not provided adequate information on current mood symptoms or on diagnosis (current, past or never) or treatment of depression, as their depression status could not be classified. The final sample for analysis included 391 VITAL-DEP participants.

### Ethics statement

We obtained written, informed consent from all participants. This study received Human Subjects research approval from the Institutional Review Board (IRB) at Partners HealthCare-Brigham and Women’s Hospital.

### Assessment of physical activity

PA was ascertained via self-report on VITAL questionnaires. Participants completed a physical activity grid and reported their average total time per week engaged in specific types of physical activity, including low-intermediate and vigorous intensity activities. To quantify intensity of PA, each activity was assigned a metabolic equivalent of task (MET) value based on a compendium of physical activities [27]. MET-hours/week was derived for each activity by multiplying its assigned MET value by the participant’s reported average number of hours per week in that activity; the total value for MET-hours/week also included the average number of flights of stairs climbed daily.

Frequency of PA was defined as the sum of reported hours spent per week in any form of PA. Type of PA was also classified: 1) low-to-intermediate intensity PA, defined as the sum of hours spent per week in yoga, stretching, walking, bicycling, weight lifting, and other exercise; 2) vigorous intensity PA, defined as the sum of hours spent per week in running, jogging, lap swimming, tennis and intense aerobics. The PA questionnaire has been previously validated among men and women of similar ages [28, 29]. Prior validation work indicated that the PA questionnaire is reproducible and provides a useful measure of average weekly activity over a 1-year period. To further address validity of self-reported PA within our own sample, we computed its Spearman rank correlation with body mass index (BMI) and observed a negative correlation (Spearman rho (ρ) = -0.29, p: <0.001). We also calculated scores on the 10-item Physical Functioning (PF-10) scale from the Medical Outcomes Short Form (SF)-36 questionnaire [30, 31], to determine its correlation with PA as an additional validity check. The PF-10 is a validated measure of physical functioning [31]; it has been used in other cohort studies to evaluate lifestyle/behavioral predictors of healthy aging among older men and women [32–35] as well as to develop a comprehensive medical comorbidity index among older adults [36]. In our study sample, we observed a positive Spearman rank correlation between physical activity (MET-hours/week) and the physical function score (ρ = 0.33, p: <0.001).

### Assessment of alcohol consumption

Frequency of alcohol consumption was ascertained via self-report on the VITAL questionnaires. Determination of alcohol consumption was based on responses to questions on the frequency of intakes of beer, wine and/or liquors; frequency of alcohol consumption was grouped into four categories–rare/never, monthly, weekly and daily use.

### Assessment of cigarette smoking

Cigarette smoking was calculated as total smoking pack-years, based on responses in the VITAL questionnaires. If participants were current or past smokers, then smoking pack-years was calculated by multiplying responses to the questions on the average cigarettes/day smoked (1 pack = 20 cigarettes) and total years of smoking; participants who never smoked were assigned to 0 pack-years. Smoking was categorized into the following groups: 0, > 0–4.99, 5.0–29.99, ≥ 30 pack-years.

### Assessment and measures of depression

The primary aims of the VITAL-DEP ancillary study were to ascertain risk of incident and recurrent depression among those without depression at baseline [24]. Briefly, depression status was ascertained using information on depressive symptoms, diagnosis and treatment data, consistent with our prior work [37] and other large-scale, high quality studies of older adults [38, 39]. The Patient Health Questionnaire-8 (PHQ-8) was used to assess mood; it has high sensitivity and specificity for depression (PHQ-8 ≥ 10) [40], validity for identifying major depression [41], and cross-cultural validity among diverse samples of older adults [42, 43]. Participants were characterized as having: no prior history of depression (i.e., eligible for incidence); past depression, but not active in the last 2 years (i.e., eligible for recurrence); prevalent depression (current clinical symptoms, diagnosis and/or treatment of depression).

### Assessment of covariates

Demographic characteristics included: age (in years), sex (men/women), racial/ethnic groups (self-reported non-Hispanic White, Black, and Hispanic), education (<high school, high school diploma, attended or graduated from college, and post-college) and annual household income (< $15,000, $15,000–49,999, $50,000–89,999, $90,000–120,000 and > $120,000). BMI was calculated as weight in kilograms divided by the square of height in meters and was categorized as normal (18.5–24.9), overweight (25.0–29.9) and obese (≥ 30). Health-related variables included: self-reported multivitamin use and health related variables such as history of diagnosis or treatment for hypertension, diabetes and current use of cholesterol-lowering medication.

### Mitochondrial DNA copy number assay and quality control

We used a quantitative PCR (qPCR) assay [44, 45], which involves a multiplex ND2 (single-copy mitochondrial gene) and AluYb8 (nuclear repeat element) PCR reaction mixture, to measure relative mtDNAcn. The qPCR-based assay determined the mitochondrial ND2 gene copy number to genomic single-copy gene copy number (N/S) ratio, a value proportional to the average mtDNAcn. Detailed procedures for the mtDNAcn assay are described elsewhere [46]. To evaluate reliability of the mtDNAcn assay, we assessed quality control (QC) by including 24 blinded samples (measured in triplicate), with random placement on the plates. To avoid batch variation effects and to reduce random variability, we ran samples in a single batch. The average coefficient of variation in the QC samples was 7.0%.

### Statistical analyses

Because the distribution of mtDNAcn was right-skewed, it was normalized using log-transformation before analysis. We evaluated associations of lifestyle and behavioral factors with log-mtDNAcn using multivariable linear regression models. Total PA was assessed as both a continuous (MET-hours/week) and categorical variable (0–4.99, 5–29.99 and ≥ 30 MET-hours/week). Frequency (hours/week of any exercise) and type (i.e., low-to-intermediate or vigorous intensity) of PA were used as categorical predictors. Cigarette smoking was assessed as a continuous variable (per 5 smoking pack-years) and categorical variable (0, > 0–4.9, 5.0–29.9, ≥ 30 pack-years); furthermore, using a cut-point based on the distribution and prior literature [47], we examined potential threshold associations by comparing mtDNAcn in categories of heavy smokers (≥ 30 pack-years) vs. all lower smoking categories (< 30 pack-years). Alcohol consumption and depression history were included as categorical variables (for alcohol: daily and less frequent than daily use; for depression: past, current and no history of depression). Estimates for the relations of age, sex and race/ethnicity to log-mtDNAcn were obtained using the multivariable linear regression models. We used multiplicative interaction terms to test possible effect modification by age, sex and race/ethnicity in the associations of lifestyle and lifestyle/behavioral factors with log-mtDNAcn. In addition, we planned stratified analyses to follow-up on evidence of interactions.

Age was modeled as a continuous variable (in years). For linear trend testing and to increase interpretability, the z-score of continuous PA MET-hours/week was used. For analyses using categorical exposures we used the following reference categories: income of < $50,000, less than post-college education, normal weight BMI category (18.5–24.9 kg/m^2^), lower PA level (intensity < 5 MET-hours/week; amount/frequency < 1 hour/week, low-intermediate and vigorous intensity exercise type < 0.75 hours/week), less than daily alcohol use frequency (i.e., rarely/never, monthly or weekly), smoking < 30 pack-years, and no history of depression. The PA and smoking exposures were entered into models as continuous variables to evaluate linear, or dose-response, associations with mtDNAcn, and separately entered into models as high vs. lower categories to assess threshold associations. Regression models were initially adjusted for age and sex (model 1), and additionally adjusted for race/ethnicity, education, income, BMI categories, PA, alcohol, smoking, and depression case status (model 2). A final regression model further adjusted for health-related variables described above (model 3). For models 2 and 3, missing indicators were created for missing covariates. We examined the association between each key exposure and log-mtDNAcn while including the other above-listed exposures as potential confounders. For example, when examining the relationship between alcohol and log-mtDNAcn, we included only non-alcohol exposures as confounders; the same approach was used when addressing PA, smoking and depression measures as exposures. Given that the dependent variable mtDNAcn was log-transformed, the regression coefficients (betas, or b) were exponentiated using the formula 100% x (e^b^ -1), with the 95% CIs estimated as 100% x (e^(b ± 1.96 x SE)^-1) and presented as percent differences in mtDNAcn.

We also conducted secondary analyses. First, we addressed obesity, which is a health status that can result from combined influences of genetic and/or lifestyle/behavioral factors (e.g., physical inactivity, unhealthy or calorie-dense diet) and may accelerate the aging process through various biological mechanisms including mitochondrial dysfunction [48, 49]. Using multivariable regression analyses, we evaluated the association between BMI and log-mtDNAcn and whether these associations were influenced by age, sex and/or race/ethnicity. BMI was assessed as both a continuous and categorical variable (overweight, obese vs. normal). Second, to increase the ability to compare our results with the existing literature regarding the relationships of different aging markers with chronological age and also with each other [20–22], we examined correlations of mtDNAcn with chronological age and another molecular aging marker, relative telomere length (RTL) (also measured by qPCR method; see elsewhere for details [21]), among a sub-set (n = 277) of our participants who had both mtDNAcn and RTL measures. Third, to observe the combined effect estimates for the association of current depressive symptoms (measured by PHQ-8 score) and past history of depression with log-mtDNAcn, we ran regression analysis with an interaction term of mean-centered PHQ-8 score and past depression; participants with current/prevalent depression were excluded for this analysis.

All statistical analyses were performed with SAS version 9.3 (SAS, Cary, NC). Statistical significance was determined using a two-tailed p-value with alpha level <0.05 for all analyses.

## Results

The sample included 391 participants [mean (standard deviation (SD)) age = 67.2 (7.7) years; 49.9% women]. There were 183 (46.8%) Non-Hispanic White, 110 (28.1%) Black and 98 (25.1%) Hispanic participants. Table 1 provides descriptive statistics grouped by race/ethnicity. Hispanic participants had slightly older age and lower representation of women than other racial/ethnic groups. Non-Hispanic Whites had higher income and post-baccalaureate education levels compared to other groups. Black participants reported lower levels of PA than other groups. Black and Hispanic adults had higher BMI, lower smoking pack-years and were less likely to consume alcohol on daily basis; non-Hispanic Whites had more than three times the prevalence of daily alcohol consumption compared to Black participants (29.0% vs 9.1%). Regarding health-related factors, Black participants had twice the prevalence of diabetes compared to non-Hispanic Whites (23.6% vs 12.6%). Average PHQ-8 score was similar across groups, but Black and Hispanic participants had higher prevalence of past depression compared to non-Hispanic Whites.

**Table 1:** Baseline characteristics of the study sample by racial/ethnic groups*.

Characteristic	Total cohort^a^	Non-Hispanic White	Black	Hispanic
	(n = 391)	(n = 183)	(n = 110)	(n = 98)
Age in years, mean (SD)	67.2 (7.7)	66.3 (7.5)	67.1 (8.0)	68.9 (7.2)
Women, %	49.9	53.6	49.1	43.9
Post-college education, %	44.5	50.3	41.8	36.7
Income ≥ 50,000 per year, %	64.7	74.9	52.0	60.0
BMI, Mean ± SD	28.0 (5.4)	26.6 (4.5)	29.9 (6.0)	28.4 (5.4)
BMI categories, kg/m^2^, %				
Normal (18.5–24.9)	30.3	41.2	12.5	28.9
Overweight (25.0–29.9)	42.0	39.0	47.1	42.3
Obesity (30+)	27.7	19.8	40.4	28.9
PA, MET-hours/week, median (IQR)^b^	18.4 (6.0–34.4)	21.4 (10.0–40.4)	8.9 (2.8–25.6)	19.8 (5.5–42.9)
Frequency of PA^c^, hours/week, %				
< 1.00	20.5	11.5	33.6	22.5
1.00 to 3.49	25.6	28.4	23.6	22.5
3.50 to 6.99	22.5	26.2	19.1	19.4
7+	31.5	33.9	23.6	35.7
Low-to-intermediate intensity PA^d^, hours/week, %				
<0.75	20.0	10.9	31.8	23.5
0.75 to 1.99	18.4	18.6	20.9	15.3
2.00 to 4.99	22.3	26.2	19.1	18.4
5+	39.4	44.3	28.2	42.9
Vigorous intensity PA^e^, hours/week, %				
<0.50	58.3	55.7	61.8	59.2
0.50 to 1.49	16.1	14.2	19.1	16.3
1.50 to 3.00	14.8	16.4	13.6	13.3
3+	10.7	13.7	5.5	11.2
Heavy cigarette smokers (≥ 30 smoking pack-years)^f^, %	5.9	7.7	2.7	6.1
Daily alcohol use, %	20.2	29.0	9.1	16.3
Multivitamin use, %	42.6	42.6	40.0	45.4
Hypertension, %	49.2	41.2	65.5	45.8
Diabetes, %	17.7	12.6	23.6	20.4
Cholesterol lowering medication, %	38.1	41.5	32.1	38.5
Median (IQR) PHQ Score	1.0 (0.0–2.0)	1.0 (0.0–2.0)	1.0 (0.0–2.0)	0.0 (0.0–1.0)
Depression status^g^, %				
No prior history of depression	77.5	70.0	82.7	85.7
Past, but not current, depression	8.7	7.1	10.9	9.2
Prevalent depression	13.8	23.0	6.4	5.1
Median (IQR) mtDNAcn	0.49 (0.33–0.67)	0.49 (0.33–0.69)	0.52 (0.33–0.74)	0.47 (0.34–0.62)

Abbreviations–PA–Physical Activity; MET—Metabolic Equivalent of Task; PHQ8—Patient Health Questionnnaire-8; BMI—Body Mass Index; mtDNAcn–Mitochondrial DNA Copy Number; SD—Standard Deviation; IQR—Inter Quartile Range

* Figures for percentages may not add to 100.0 due to rounding.

^a^ For normally distributed continuous variables, this column contains mean (standard deviation) for non-missing responses. For non-normally distributed continuous variables, this column contains median (interquartile range) and percentages for categorical variables.

^b^ Total physical activity was based on amount of reported time spent per week in the physical activities and reported number of flights of stairs climbed daily.

^c^ Leisure-time physical activities: yoga / stretching / toning, walking, bicycling, weight-lifting/strength training, jogging, running, aerobic exercise/aerobic dance/exercise machines, tennis/squash/ racquetball, lap swimming, other exercise.

^d^ Low-to-intermediate intensity physical activity includes walking, yoga / stretching / toning, bicycling, weight-lifting and other exercises.

^e^ Vigorous intensity physical activity includes running, jogging, lap swimming, tennis, squash/racquetball, intense aerobics.

^f^ Tobacco smoking was measured as smoking pack-years (average cigarettes per day / 20 * total years smoked). Never smokers were considered as having zero smoking pack-years.

^g^ Depression status categories were characterized based on no prior history of depression (i.e. eligible for incidence); or had prior history of depression, but not active in the last 2 years (i.e., eligible for recurrence); or prevalent case of depression (any of current clinical symptoms, diagnosis and/or treatment of depression).

Regarding associations of age, sex and race/ethnicity with log-mtDNAcn, we did not observe any significant percent difference in mtDNAcn for each year increase of age (S1 Table), and there were no meaningful differences in mtDNAcn across racial/ethnic groups; however, there was a significant positive association of higher mtDNAcn among women compared to men (percent difference = 16.9%, 95% CI = 3.6%, 32.0%) (S1 Table). Of note, as our sample was a sub-set of a larger cohort, we compared baseline characteristics between the selected 450 participants and the full VITAL cohort to verify they were comparable. Comparing the selected 450 participants vs. the full VITAL cohort: mean (SD) age: 67.1 (7.7) vs. 67.1 (7.1) years; mean (SD) BMI: 28.0 (5.4) vs. 28.1 (5.7) kg/m^2^; post-baccalaureate education: 44.4% vs. 45.2%. Additionally, characteristics of the final sample (n = 391) were the same as those of the initially selected participants (n = 450); e.g., comparing these respective groups: mean (SD) age: 67.2 (7.7) vs. 67.1 (7.7) years; mean (SD) BMI: 28.0 (5.4) vs. 28.0 (5.4) kg/m^2^ (i.e., same in both groups); post-baccalaureate education: 44.5% vs. 44.4%.

Table 2 shows results for percent differences in mtDNAcn across various PA measures. We did not observe a linear association between continuous PA (per 1-SD change, where 1-SD = 26.2 MET-hours/week) and mtDNAcn. Similarly, when addressing categories of PA intensity and frequency, there was no evidence of a dose-response association between the amount of PA and mtDNAcn (non-significant p-trends), and we did not observe any differences in mtDNAcn across different types of PA (i.e., low-to-intermediate or vigorous intensity). In addition, there were no variations by age, sex or race/ethnicity in the associations between amount or type of PA and mtDNAcn (all p-interactions were non-significant).

**Table 2:** Association between physical activity measures and percent differences (95% CI) in mitochondrial DNA copy number (log-transformed N/S ratio).

Exposures*	Model 1^d^	Model 2^e^	Model 3^f^
***Total Physical Activity***^***a***^**, *MET-hours/week*, *continuous***			** **
Per 1 SD, *n = 391*	-1.6 (-7.1, 4.1)	-1.0 (-6.7, 5.1)	-1.8 (-7.5, 4.3)
***Total Physical Activity*, *MET-hours/week***			
<5, *n = 91*	Ref	Ref	Ref
5 to 29.99, *n = 189*	9.3 (-5.4, 26.3)	10.1 (-5.2, 27.8)	9.5 (-5.7, 27.1)
≥30, *n = 111*	-4.4 (-18.6, 12.4)	-3.2 (-18.5, 15.1)	-4.2 (-19.5, 13.9)
***Frequency of Physical Activity*, *hours/week***			
<1, *n = 80*	Ref	Ref	Ref
1 to 3.49, *n = 100*	-4.8 (-19.4, 12.5)	-5.4 (-20.5, 12.6)	-3.1 (-18.6, 15.5)
3.50 to 6.99, *n = 88*	16.3 (-2.1, 38.2)	14.2 (-4.5, 36.6)	13.5 (-5.1, 35.9)
7+, *n = 123*	-7.7 (-21.4, 8.5)	-6.9 (-21.7, 10.5)	-6.8 (-21.5, 10.8)
***Low-to-intermediate intensity activities***^***b***^**, *hours/week***			
<0.75, *n = 78*	Ref	Ref	Ref
0.75 to 1.99, *n = 72*	-11.2 (-26.1, 6.6)	-13.8 (-28.3, 3.7)	-12.4 (-27.3, 5.5)
2 to 4.99, *n = 87*	-2.6 (-18.3, 16.1)	-4.4 (-20.2, 14.6)	-4.5 (-20.4, 14.5)
5+, *n = 154*	-3.2 (-17.3, 13.2)	-3.5 (-18.0, 13.7)	-3.8 (-18.4, 13.4)
***Vigorous intensity activities***^***c***^**, *hours/week***			
<0.5, *n = 228*	Ref	Ref	Ref
0.50 to 1.49, *n = 63*	5.8 (-9.9, 24.3)	3.9 (-11.7, 22.3)	3.7 (-11.9, 22.1)
1.50 to 2.99, *n = 58*	-3.7 (18.3, 13.7)	-4.8 (-19.3, 12.4)	-4.5 (-19.2, 12.8)
3+, *n = 42*	-5.0 (-21.5, 14.9)	-3.3 (-20.4, 17.3)	-5.7 (-22.5, 14.7)

Abbreviations: SD–Standard Deviation.

* All p-trends across PA exposures were > 0.05, calculated for model 3. Relationship of total PA and log-mtDNAcn was assessed for the interactions of age, sex and race/ethnicity. We did not observe any significant variations for these relationships by age, sex or race/ethnicity (all p-interactions > 0.05).

^a^ Total physical activity was based on amount of reported time spent per week in the physical activities and reported number of flights of stairs climbed daily.

^b^ Low-to-intermediate intensity physical activity includes walking, yoga / stretching / toning, bicycling, weight-lifting and other exercises.

^c^ Vigorous intensity physical activity includes running, jogging, lap swimming, tennis, squash/racquetball, intense aerobics.

^d^ Model 1 was adjusted for age (years) and sex.

^e^ Model 2 was adjusted for age (years), sex, race, education (post-college vs college or under), income (<$50,000 vs ≥ $50,000), body mass index categories (18.5–24.9, 25.0–29.9, ≥ 30 kg/m^2^), alcohol consumption (daily vs less frequent than daily), smoking use (<30 vs ≥ 30 smoking pack-years) and depression category (past but not current history of depression or prevalent depression vs no prior history of depression)

^f^ Model 3 was adjusted for age (years), sex, race, education (post-college vs college or under), income (<$50,000 vs ≥ $50,000), body mass index categories (18.5–24.9, 25.0–29.9, ≥ 30 kg/m^2^), alcohol consumption (daily vs less frequent than daily), smoking use (<30 or ≥ 30 smoking pack-years) and depression category (past but not current history of depression or prevalent depression vs no prior history of depression), multivitamin use, comorbid conditions (hypertension, diabetes and use of cholesterol lowering medication).

Relations of alcohol consumption, smoking and depression variables to percent differences in mtDNAcn are shown in Table 3. There was no association between daily alcohol consumption and mtDNAcn. Higher cigarette smoking was significantly associated with lower mtDNAcn in the fully adjusted model: each 5 pack-year increment of cigarette smoking was associated with an average 2.9% (95% CI = -5.4%, -0.4%) lower mtDNAcn. Furthermore, in analyses of pack-years as categorical variables, we observed a threshold association such that persons with heavy smoking (≥30 pack-years) had significantly lower mtDNAcn compared to those who never smoked (percent difference = -28.5%; 95% CI = -44.2%, -8.3%). Regarding depression variables, there was suggestive difference of lower mtDNAcn among participants with vs. without past depression history (percent difference = -15.1%; 95% CI = -30.8%, 4.1%), but this was not statistically significant. We also did not observe a significant difference in mtDNAcn between those with prevalent depression vs. no history of depression (percent difference = 5.0; 95% CI = -12.3%, 25.8%).

**Table 3:** Associations between cigarette smoking, alcohol consumption, and depression and percent differences (95% CI) in mitochondrial DNA copy number (log-transformed N/S ratio).

Exposures*	Model 1^a^	Model 2^b^	Model 3^c^
***Alcohol Consumption***			
Less frequent than daily use, *n = 312*	Ref	Ref	Ref
Daily use, *n = 79*	-7.9 (-20.2, 6.3)	-7.1 (-19.9, 7.8)	-6.6 (-19.6, 8.4)
***Cigarette smoking (pack-years)–continuous***	** **	** **	** **
Per 5 pack-years, *n = 391*	-2.6 (-5.0, -0.1)	-3.0 (-5.4, -0.5)	-2.9 (-5.4, -0.4)
***Cigarette smoking (pack-years)–categorical***			
0, *n = 204*	Ref	Ref	Ref
> 0–4.99, *n = 70*	0.8 (-13.6, 17.7)	3.4 (-11.7, 21.1)	2.8 (-12.1, 20.3)
5–29.99, *n = 94*	2.0 (-11.3, 17.2)	-0.0 (-13.3, 15.3)	1.2 (-12.3, 16.8)
30+, *n = 23*	-27.1 (-42.9, -6.9)	-28.5 (-44.2, -8.3)	-28.5 (-44.2, -8.3)
***Depression status***	** **	** **	** **
No prior history of depression, *n = 303*	Ref	Ref	Ref
Past, but not current, depression, *n = 34*	-14.4 (-30.3, 5.2)	-14.4 (-30.2, 5.1)	-15.1 (-30.8, 4.1)
Prevalent depression, *n = 54*	0.3 (-15.7, 19.4)	3.5 (-13.5, 23.8)	5.0 (-12.3, 25.8)

* P values for per 5 smoking pack-years: 0.02; for ≥ 30 smoking pack-years: 0.009; for past, but not current depression: 0.12. All other p-values were non-significant and calculated based on Model 3. Relationships between alcohol consumption, cigarette smoking, and depression status with log-mtDNAcn were assessed for the interactions of age, sex and race/ethnicity. We did not observe any significant variations for these relationships (all p-interactions > 0.05) except for cigarette smoking. When assessing the interaction between smoking (per 5 pack-years), the p-interaction was 0.02 for sex and 0.01 for race/ethnicity.

^a^ Model 1 was adjusted for age (years) and sex.

^b^ Model 2 was adjusted for age (years), sex, race, education (post-college vs college or under), income (<$50,000 vs ≥ $50,000), body mass index categories (18.5–24.9, 25.0–29.9, ≥ 30 kg/m^2^), total physical activity (<5, 5–29.99, ≥ 30 MET-hours/week), alcohol consumption (daily vs less frequent than daily), smoking use (<30 vs ≥ 30 smoking pack-years) and depression category (past but not current history of depression or prevalent depression vs no prior history of depression)

^c^ Model 3 was adjusted for age (years), sex, race, education (post-college vs college or under), income (<$50,000 vs ≥ $50,000), body mass index categories (18.5–24.9, 25.0–29.9, ≥ 30 kg/m^2^), total physical activity (<5, 5–29.99, ≥ 30 MET-hours/week), alcohol consumption (daily vs less frequent than daily), smoking use (<30 or ≥ 30 smoking pack-years) and depression category (past but not current history of depression or prevalent depression vs no prior history of depression), multivitamin use, comorbid conditions (hypertension, diabetes and use of cholesterol lowering medication).

**Fig 1:**
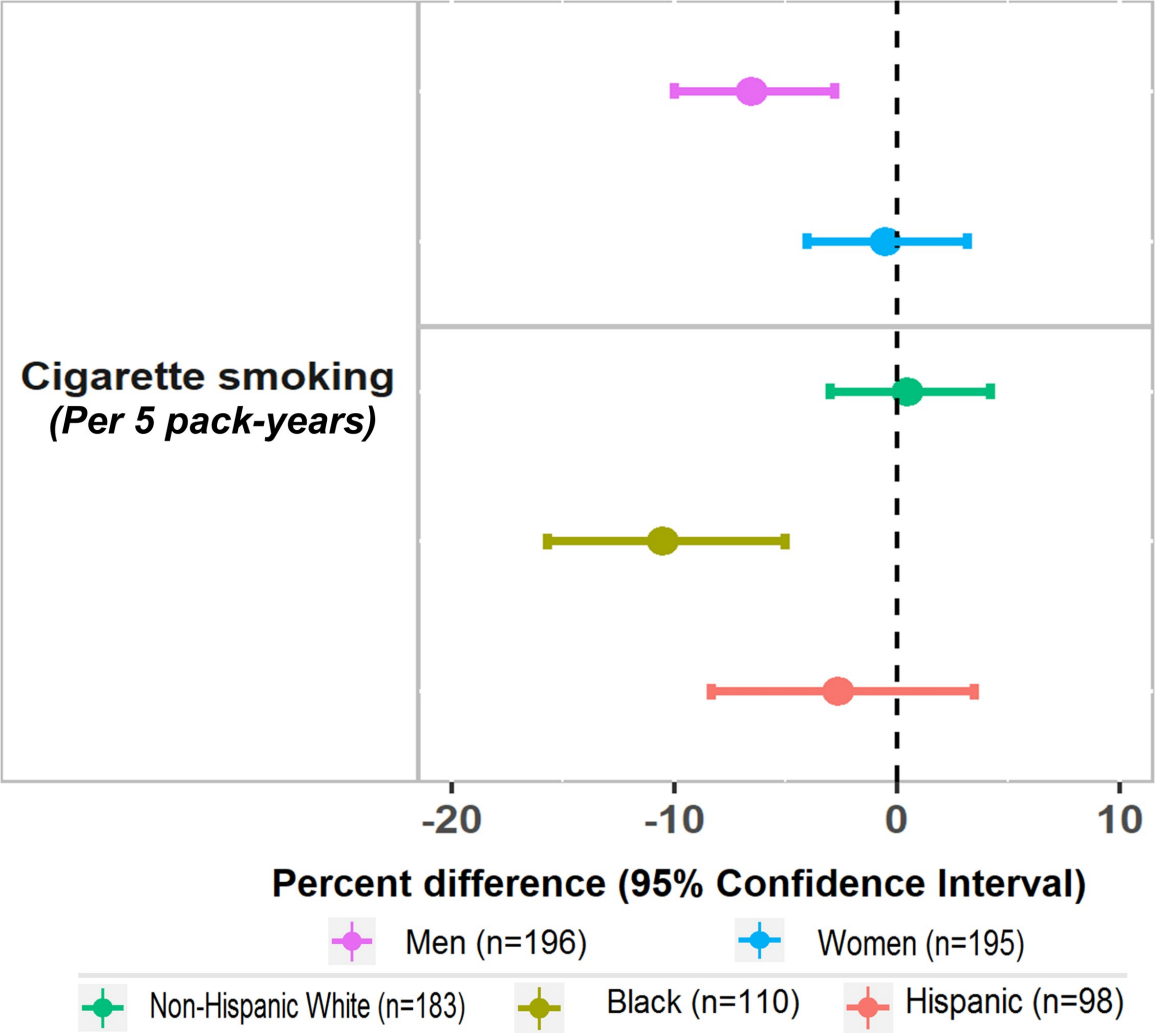
Association between cigarette smoking (per 5 pack-years) and percent differences (95% CI) in mitochondrial DNA copy number (log-transformed N/S ratio), stratified by sex and race/ethnicity. * Estimates were calculated based on model adjusted for age (years), sex, race, education (post-college vs college or under), income (<$50,000 vs ≥ $50,000), body mass index categories (18.5–24.9, 25.0–29.9, ≥ 30 kg/m^2^), total physical activity (<5, 5–29.99, ≥ 30 MET-hours/week), alcohol consumption (daily vs less than frequent daily), smoking use (<30 or ≥ 30 smoking pack-years) and depression category (past but not current history of depression or prevalent depression vs no prior history of depression), multivitamin use, comorbid conditions (hypertension, diabetes and use of cholesterol lowering medication).

Regarding interactions, we observed significant variations by sex and race/ethnicity for the association between cigarette smoking (per 5 pack-years) and mtDNAcn. However, there was no evidence of variation by age, sex or race/ethnicity in the associations of any other lifestyle or behavioral factors with mtDNAcn. In analyses that further stratified participants by sex and race/ethnicity, there was an association between smoking and significantly lower mtDNAcn in men but not women; we also observed an association between smoking and significantly lower mtDNAcn among Black participants but not among non-Hispanic White or Hispanic adults (Fig 1).

Finally, to address robustness of results and to place them in the context of existing literature, we ran additional analyses. First, in the secondary analysis addressing BMI as a predictor, we did not observe any significant overall association between BMI and mtDNAcn (S2 Table). However, we observed potential evidence of meaningful variation by sex for the association between obesity (≥ 30 kg/m^2^) and mtDNAcn (p-interaction = 0.06). In a sex-stratified analysis, there was an association between obesity and higher mtDNAcn among men, but not in women (percent difference (95% CI): for men = 42.9 (10.6, 84.6); for women = -7.1 (-26.7, 17.6)). Second, we addressed correlations of mtDNAcn with chronological age and telomere length among the two-thirds of participants (n = 277) who had both mtDNAcn and RTL measures. Due to the skewed distributions of these biomarkers, we used Spearman rank correlations to estimate the correlation coefficients conservatively. While mtDNAcn was not correlated with chronological age (ρ = 0.04, p = 0.47), there was a borderline negative correlation observed between RTL with chronological age (ρ = -0.11, p = 0.06) (S2 Fig). We did not observe a significant correlation between RTL and mtDNAcn (ρ = -0.09, p = 0.13). Third, we addressed the combined influence of current depressive symptoms and past history of depression on log-mtDNAcn. Among those with past depression history, there were no differences in mtDNAcn comparing those with vs. without current severe depressive symptoms (p-interaction = 0.74).

## Discussion

In this cross-sectional study that included a diverse sample of 391 community-dwelling mid-life and older adults, we observed varying associations of lifestyle and behavioral factors with mtDNAcn. Cigarette smoking was significantly associated with mtDNAcn. There was mean 2.9% lower mtDNAcn for every 5 pack-year increment of cigarette smoking. Furthermore, there appeared to be a threshold association for smoking, such that estimated mtDNAcn was significantly lower among those with ≥ 30 pack-years compared to those who have never smoked and all those below the 30 pack-year smoking level. By contrast, no statistically significant associations were observed between other lifestyle and behavioral factors–including type, amount or intensity of PA, alcohol consumption or depression status–with mtDNAcn. We observed significant variations by sex and race/ethnicity in the association between smoking and mtDNAcn, but not in associations between other lifestyle/behavioral factors and mtDNAcn. In stratified analyses, statistically significant associations between higher smoking pack-years and lower mtDNAcn were more apparent among men and Black participants.

We hypothesized protective associations between higher PA and mtDNAcn. In our sample, we did not observe any significant association of mtDNAcn with total PA. The existing literature regarding associations between PA and mtDNAcn is mixed. For example, in a prior cross-sectional study that evaluated PA among 144 post-menopausal women, higher mtDNAcn was observed among those who did vs. did not regularly participate in moderate and/or vigorous intensity exercise [50]. By contrast, in a case-control study that included participants with colorectal cancer and healthy controls, there was no association between moderate or vigorous weekly PA and mtDNAcn among n = 874 healthy controls; mtDNAcn mean values were identical among those who did vs. did participate in weekly exercise [51]. Compared to prior work, our study is noteworthy as it specifically addressed how different PA measures (frequency, amount or intensity) relate to mtDNAcn. Taken together with other reports, our findings suggest a need for further investigation, including studies addressing mechanisms and different age, sex and/or race/ethnicity groups, of a likely complex association between PA and mtDNAcn. For example, the amount and intensity of PA may have variable associations with regulation of mtDNAcn and/or mutations in the mtDNA genome [52], and such factors may play a role in the conflicting reports regarding associations between PA and mtDNAcn.

The results for the relationship between cigarette smoking and mtDNAcn were more consistent with extant literature [4, 12, 47]. For example, among 2,769 older women in the Nurses’ Health Study (NHS) [12] there were inverse associations of both cigarette pack-years and duration (total years) of smoking with mtDNAcn (p-trends = 0.01 and 0.007, respectively); consistent with results from our study, there was a negative association between pack-years and mtDNAcn. Lee and colleagues [47] evaluated potential associations between smoking and alteration in the relative mtDNA content in human lung. Although the biospecimen source used in the Lee et al. study differed from that in our VITAL-DEP sample (i.e., lung vs. peripheral blood leukocytes), results were consistent with our findings: the average content of mtDNA in the lung tissues of older adults with heavy smoking (≥ 30 pack-years) was significantly lower than that in persons with lower smoking (< 30 pack-years) (p < 0.05). Regarding alcohol consumption, there have been few studies and mixed results have been reported for the magnitude of associations between alcohol consumption and mtDNAcn [22, 53]. Finally, results from prior studies of mid-life to older adults examining associations between depression outcomes (e.g., diagnosis of major depressive disorder or depressive symptom levels) and mtDNAcn have been mixed; reported associations were positive [16, 54], inverse [13, 55] or null [15]. In our study, non-statistically significant estimates were in the direction of lower mtDNAcn with past depression. However, there were few participants who were past depression cases; thus, power was lower to detect whether the association between past depression with mtDNAcn differed meaningfully by sex or race/ethnicity. Also, as VITAL-DEP was designed to focus on depression risk among those without prevalent depression at baseline, the range of PHQ-8 scores in this study was constricted.

Regarding variation by demographic factors, we observed that the association between cigarette smoking and mtDNAcn varied by sex and race/ethnicity. Since we observed lower average mtDNAcn in men compared to women, the stronger inverse association (seen in the stratified analyses) between smoking and mtDNAcn among men suggests the speculative possibility that the health penalty of smoking may be more severe for this group. Similarly, in the analyses stratified by race/ethnicity, the negative association between smoking and mtDNAcn was stronger among Black participants compared to non-Hispanic white or Hispanic adults. Notably, such results would not have been observed in prior large cohort studies that had included only women participants and/or were not racially/ethnically diverse. Thus, these novel findings suggest important new biological paths to explain health disparities, as they raise the possibility of differential adverse impacts of modifiable behaviors across different demographic groups. To that end, results from our study will provide a useful basis of comparison for future work that addresses associations between lifestyle/behavioral factors and molecular markers of oxidative stress or biological aging in racially/ethnically diverse samples of men and women.

In a secondary analysis, we observed no significant differences in mtDNAcn among overweight and obese participants compared to those with normal weight/BMI. Prior studies have reported negative associations between obesity and mtDNAcn [22, 56]. Those studies were conducted among participants with notable differences in sample characteristics compared to this study. For example, a study by Meng et al. [22] included 1700 mostly non-Hispanic white older women (mean age at blood draw: 58 y), and another study by Lee et al. [56] was conducted among 94 healthy young adults [mean age: 29.6 y; women: n = 40 (42.6%)]. Participants in our study were older and balanced by sex. Furthermore, this study was conducted in the context of evaluating potential sources of health disparities among racial/ethnic groups; thus, there was larger representation of minority participants. Finally, regarding the preliminary suggestions of potential interactions by sex of the association between obesity and mtDNAcn (i.e., estimates for a positive association among men but not women), longitudinal investigation is required to confirm sex-specific associations.

We had hypothesized that the selected modifiable lifestyle/behavioral factors would be related to mtDNAcn because of known associations between these factors and oxidative stress and metabolic health. Indeed, mtDNAcn is correlated with mitochondrial function, energy metabolism and oxidative stress indices [57–59]; for example, a study by Wang and colleagues [58] found that markers of oxidative stress and antioxidant defense, such as thiobarbituric acid-reactive substances (TBARS), were positively correlated with mtDNAcn (p = 0.02). Our study did not include concurrent measurement of peripheral markers of oxidative stress, but there were measures of telomere length in a subset. It was noteworthy that telomere length was negatively correlated with chronological age in our sample, but mtDNAcn was not correlated with chronological age or RTL. Thus, although declining quantity of mitochondria is considered an aspect of advancing age, there was no association between mtDNA copy number and chronological age in our sample, and this finding is consistent with prior literature showing inconsistent associations between mtDNAcn and age as well as other biomarkers of aging [20–23]. Further, mtDNA mutations, heteroplasmy (i.e., affecting mtDNA *quality* as opposed to *quantity*) and tissue specificity may have independent effects on aging processes, and these factors may influence the observed magnitude and direction of associations between mtDNAcn and age [18, 19, 60]. Overall, our results are consistent with the concept that mtDNAcn may not correspond with biological aging as strongly or consistently as other molecular markers (e.g., telomere length or DNA methylation age); rather, mtDNAcn may relate to different oxidative stress states more than biological aging *per se*.

This study had notable strengths. The cohort was well-characterized and emphasized minority representation. Also, the study extended the literature by addressing frequency, amount and types (e.g., low-to-intermediate vs. vigorous activities) of PA. The evidence regarding potential variation by sex and race/ethnicity in the association between smoking and mtDNAcn was another novel contribution. There were also limitations. First, the design was cross-sectional; a longitudinal approach would be needed to estimate the association between lifestyle and behavioral factors and actual prospective change in mtDNAcn during aging. Second, the sample included generally healthy participants without any history of cancer or CVD; however, while this may have affected generalizability, it was not expected to affect the internal validity of the findings. Third, the assessment of lifestyle/behavioral factors was self-reported, and exposure misclassification was possible; however, we used established, validated measures (e.g., use of a validated PA measure; strong correlation between PA and the physical functioning scale; use of the validated PHQ-8). Fourth, we were limited in our ability to address relations of severity of current depressive symptoms or duration of past depressive episodes to mtDNAcn. Fifth, our study did not include concurrent measurement of peripheral markers of oxidative stress (e.g., TBARS or F2-isoprostanes), and we could not confirm associations between mtDNAcn and specific oxidative stress markers in our sample. However, we measured mtDNAcn with high reliability in this study, and work by other groups previously demonstrated the strong correlation between mtDNAcn and gold-standard peripheral markers of oxidative stress.

## Conclusions

We observed a significant association between cigarette smoking and lower mitochondrial DNA copy number, but we did not observe consistent associations of other lifestyle and behavioral factors (i.e., physical activity, alcohol consumption, and depression) with mtDNAcn. Furthermore, the association between cigarette smoking and mtDNAcn varied significantly by sex and race/ethnicity; the association of smoking with lower mtDNAcn was more apparent among men and Black participants. Also, it was notable that, while the traditional biological aging marker of telomere length was correlated with chronological age in our sample, mtDNAcn was not. Thus, our results may also have implications for more nuanced considerations of mtDNAcn as an indicator of varying oxidative stress states as opposed to biological aging itself.

## Supporting information

S1 Checklist STROBE statement—Checklist of items that should be included in reports of *cross-sectional studies*. (DOCX)

S1 Fig Flow chart of study participants. Abbreviations: mtDNAcn: Mitochondrial DNA Copy Number; VITAL: VITamin D and OmegA-3 TriaL; VITAL-DEP: Depression Endpoint Prevention in VITamin D and OmegA-3 TriaL; CTSC: Clinical and Translational Center.
(TIF)

S2 Fig Correlations of chronological age with telomere length and mitochondrial DNA copy number*. *Matrix of Spearman correlation coefficients and p-values for the molecular markers and chronological age.
(TIF)

S1 Table Percent differences (95% CI) in mitochondrial DNA copy number (log-transformed N/S ratio) by demographic characteristics. (DOCX)

S2 Table Association between BMI and percent differences (95% CI) in mitochondrial DNA copy number (log-transformed N/S ratio). (DOCX)
