# Supplementary figures and images for: Lifestyle and behavioral factors and mitochondrial DNA copy number in a diverse cohort of mid-life and older adults
Source: PLoS One. 2020 Aug 12;15(8):e0237235. doi: 10.1371/journal.pone.0237235 (PMC7423118; doi:10.1371/journal.pone.0237235)

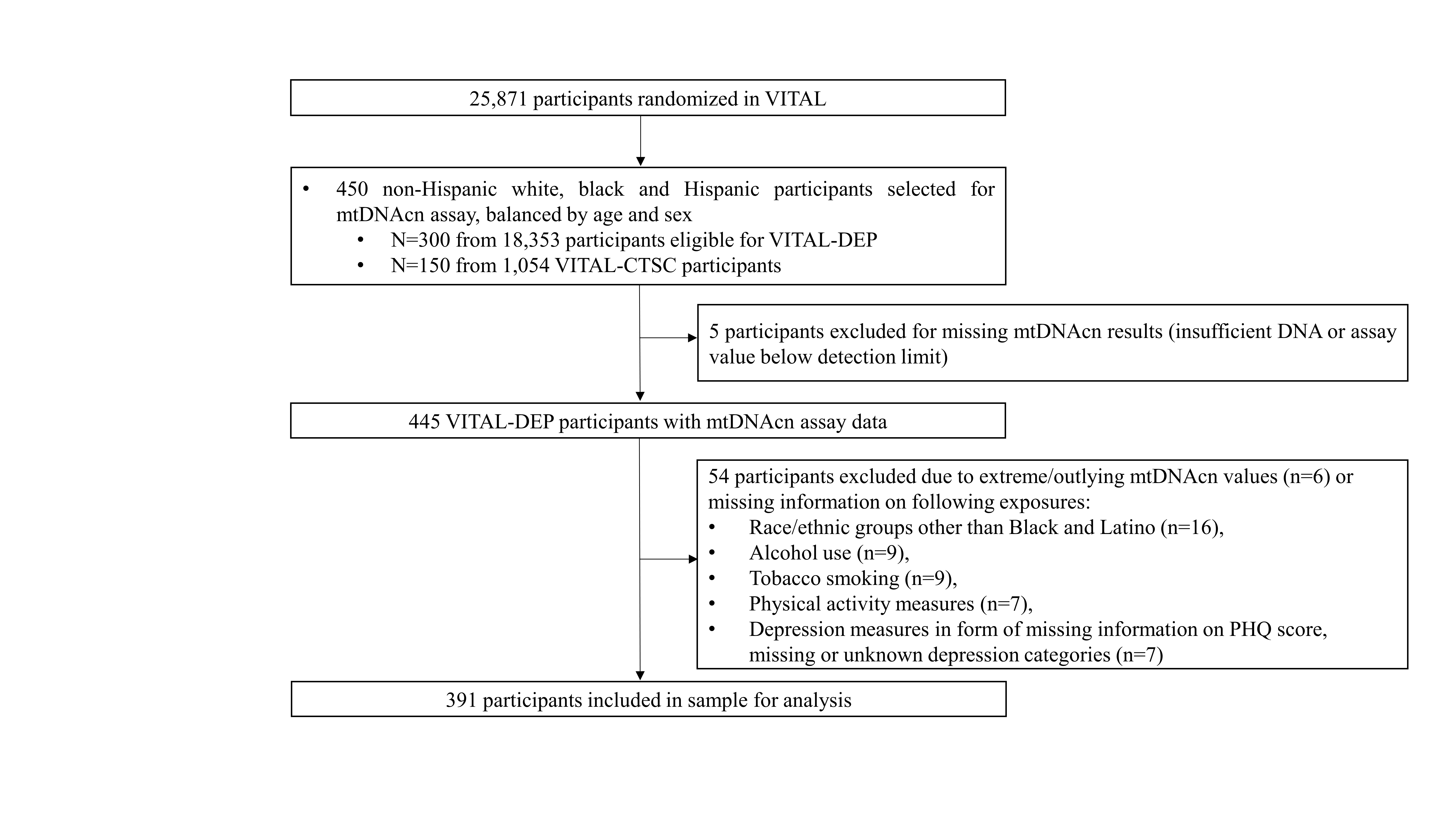

Supplement: S1 Fig — Abbreviations: mtDNAcn: Mitochondrial DNA Copy Number; VITAL: VITamin D and OmegA-3 TriaL; VITAL-DEP: Depression Endpoint Prevention in VITamin D and OmegA-3 TriaL; CTSC: Clinical and Translational Center. (TIF) [file pone.0237235.s002.tif]

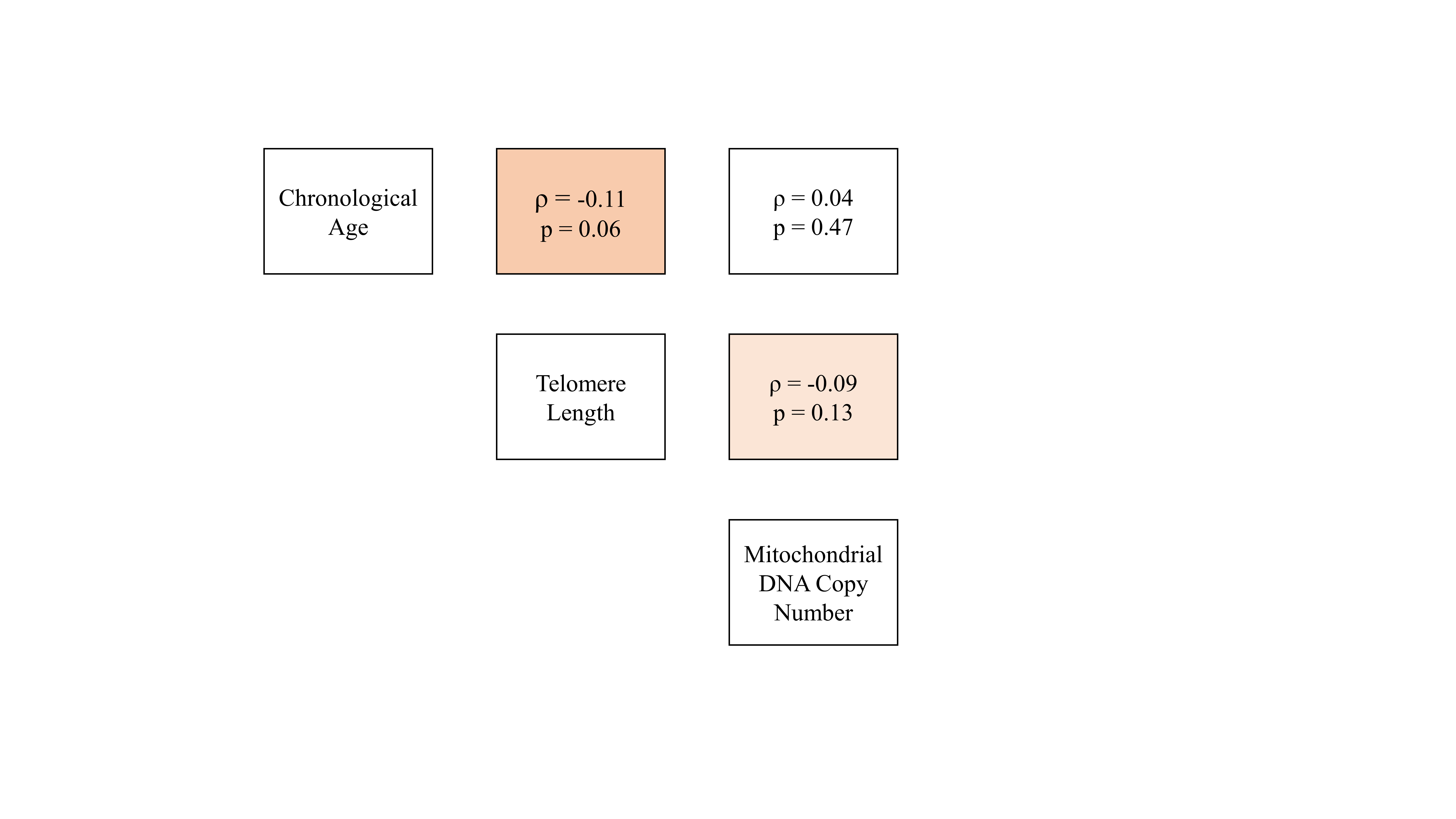

Supplement: S2 Fig — *Matrix of Spearman correlation coefficients and p-values for the molecular markers and chronological age. (TIF) [file pone.0237235.s003.tif]
