# Supplementary material for: Lifestyle and behavioral factors and mitochondrial DNA copy number in a diverse cohort of mid-life and older adults
Source: PLoS One. 2020 Aug 12;15(8):e0237235. doi: 10.1371/journal.pone.0237235 (PMC7423118; doi:10.1371/journal.pone.0237235)
Supplement: S1 Table — (DOCX) [file pone.0237235.s004.docx]

**S1 Table. Percent differences (95% CI) in mitochondrial DNA copy number (log-transformed N/S ratio) by demographic characteristics.**

| Demographic characteristics | Percent difference (95% CI)* |
| --- | --- |
| Age^a^, *n=391* | 0.3 (-0.5, 1.1) |
| Sex |  |
| Men, *n=196* | Ref |
| Women, *n=195* | 16.9 (3.6, 32.0) |
| Race/ethnicity |  |
| Non-Hispanic White, *n =183* | Ref |
| Black, *n=110* | 9.4 (-6.0, 27.5) |
| Hispanic, *n=98* | -7.6 (-20.3, 7.2) |

Abbreviation : CI – Confidence Interval

* Estimates were calculated based on model adjusted for age (years), sex, race, education (post-college vs college or under), income (<$50,000 vs ≥ $50,000), body mass index categories (18.5-24.9, 25.0-29.9, ≥ 30 kg/m^2^), total physical activity (<5, 5-29.99, ≥ 30 MET-hours/week), alcohol intake (daily vs non-daily), smoking use (<30 or ≥ 30 smoking pack-years) and depression category (past but not current history of depression, prevalent depression vs no prior history of depression), multivitamin use, comorbid conditions (hypertension, diabetes and cholesterol lowering medication).

^a^ Estimate is for a one-year difference in age.
