# Supplementary material for: Lifestyle and behavioral factors and mitochondrial DNA copy number in a diverse cohort of mid-life and older adults
Source: PLoS One. 2020 Aug 12;15(8):e0237235. doi: 10.1371/journal.pone.0237235 (PMC7423118; doi:10.1371/journal.pone.0237235)
Supplement: S2 Table — (DOCX) [file pone.0237235.s005.docx]

**S2 Table.** **Association between BMI and percent differences (95% CI) in mitochondrial DNA copy number (log-transformed N/S ratio).**

| BMI (in kg/m^2^)* | Model 1^a^ | Model 2^b^ | Model 3^c^ |
| --- | --- | --- | --- |
| BMI, continuous | 0.3 (-0.8, 1.3) | 0.4 (-0.8, 1.6) | 0.6 (-0.6, 1.7) |
| BMI categories, in kg/m^2^ |  |  |  |
| Normal (18.5 - 24.9), *n=116* | Ref | Ref | Ref |
| Overweight (25.0 - 29.9), *n=161* | 8.6 (-5.3, 24.6) | 8.5 (-5.9, 25.0) | 10.8 (-3.9, 27.9) |
| Obesity (30+), *n=106* | 8.4 (-6.8, 26.0) | 9.7 (-6.7, 29.1) | 14.4 (-3.2, 35.1) |

Abbreviation: BMI, body mass index; CI, confidence interval.

* P values for continuous BMI: 0.36; in categorical BMI - for overweight: 0.16; for obesity: 0.11. P-values were calculated based on model 3. The association between overweight and obesity with log-mtDNAcn was assessed for interactions with age, sex and race/ethnicity. We observed evidence of variation by sex for the association between obesity and log-mtDNAcn (p-interaction: 0.06). However, we did not observe any significant variations by age, sex and/or race/ethnicity for the association between overweight and log-mtDNAcn.

^a^  Model 1 was adjusted for age (years) and sex.

^b^   Model 2 was adjusted for age (years), sex, race, education (post-college vs college or under), income (<$50,000 vs ≥ $50,000), total physical activity (<5, 5-29.99, ≥ 30 MET-hours/week), alcohol consumption (daily vs less frequent than daily), smoking use (<30 vs ≥ 30 smoking pack-years) and depression category (past but not current history of depression or prevalent depression vs no prior history of depression)

^c^   Model 3 was adjusted for age (years), sex, race, education (post-college vs college or under), income (<$50,000 vs ≥ $50,000), total physical activity (<5, 5-29.99, ≥ 30 MET-hours/week), alcohol consumption(daily vs less frequent than daily), smoking use (<30 or ≥ 30 smoking pack-years) and depression category (past but not current history of depression or prevalent depression vs no prior history of depression), multivitamin use, comorbid conditions (hypertension, diabetes and use of cholesterol-lowering medication).
